# Molecularly Imprinted Polymer Nanoparticles for Lung-Cancer-Cell-Surface Proteomics

**DOI:** 10.3390/polym18020281

**Published:** 2026-01-20

**Authors:** Kirabo Magumba, Elena Piletska, Thong Huy Cao, Donald Jones, Salvador Macip, Sergey Piletsky

**Affiliations:** 1 School of Chemistry, College of Science and Engineering, University of Leicester, Leicester LE2 7RH, UK; 2 Leicester van Geest MultiOMICs Facility, University of Leicester, Leicester LE3 9QP, UK; 3 Division of Cardiovascular Sciences, College of Life Sciences, University of Leicester, Leicester LE1 7RH, UK; 4 National Institute for Health and Care Research, Leicester Biomedical Research Centre, University Hospitals of Leicester NHS Trust, Glenfield Hospital, Leicester LE3 9QP, UK; 5 Leicester Cancer Research Centre, University Hospitals of Leicester NHS Trust, Leicester Royal Infirmary, University of Leicester, Leicester LE1 5WW, UK; 6 Mechanisms of Cancer and Aging Laboratory, Division of Molecular and Cell Biology, University of Leicester, Leicester LE1 7RH, UK; 7 FoodLab, eHealth Centre, Faculty of Health Sciences, Universitat Oberta de Catalunya, 08018 Barcelona, Spain; 8 Biology of Neurodegeneration and Ageing Research Group, BarcelonaBeta Brain Research Center, Pasqual Maragall Foundation, 08005 Barcelona, Spain

**Keywords:** molecularly imprinted polymer nanoparticles, lung cancer, proteomics, protein targets

## Abstract

The identification and targeting of lung-cancer-cell-surface proteins are important for drug development. Molecularly imprinted polymer nanoparticles (nanoMIPs) offer a synthetic approach for the recognition of proteins on the cell surfaces. This work outlines the use of a novel ‘snapshot imprinting’ approach to characterize differences in the cell-surface proteomes of lung cancer cell lines (A549, H460, H522) and a non-cancerous cell line (BEAS-2B) to potential protein targets for diagnostic and therapeutic applications. The mass spectrometry-based quantitative proteomics identified 2381 proteins. Fold change and *p*-value thresholds were used to define statistically and biologically significant differentially expressed proteins (DEPs) across cell lines, yielding 353, 426, and 274 DEPs for A549, H460, and H522, respectively, when compared to BEAS-2B. The DEPs identified across overlapping cell line comparisons were analyzed using Gene Ontology enrichment and a protein–protein network to identify hub proteins. Among these hub proteins, five proteins (NPM1, TOP2A, EZH2, PRKDC, and HNRNPK) were identified as clinically relevant when cross-referenced with the Human Protein Atlas database and the literature, highlighting their potential as diagnostic and therapeutic targets. These findings highlight the potential of nanoMIP-based snapshot imprinting as an alternative to ‘classical’ approaches for identifying potential protein targets for diagnostic and therapeutic applications.

## 1. Introduction

Lung cancer is one of the most common types of cancer worldwide [1]. Despite advances in early detection and personalized medicine, the prognosis for lung cancer remains poor, particularly in the advanced stages [2]. Therefore, identifying additional molecular targets is required to develop more precise and effective diagnostic and therapeutic strategies.

Protein complexes on the cell surface constantly assemble and disassemble to regulate cellular signaling and functions. Some of these proteins shuttle between the intercellular and extracellular environments to support various biological processes, such as signal transduction, cell–extracellular matrix adhesion, and cell–cell communication [3,4,5]. Membrane proteins make up ~23% of the human proteome and account for 60% of approved drug targets; however, identifying these proteins remains challenging due to their low expression levels and hydrophobic nature [6,7].

Proteomics has emerged as an essential tool for studying the cancer cell surface proteome (surfaceome) by identifying, characterizing, and quantifying proteins. This technology enables the identification of key cell surface proteins, providing insight into the molecular mechanisms underlying cancer progression [8]. Analysis of cell surface proteins and the identification of molecular markers are crucial in the identification of diagnostic and therapeutic targets for personalized cancer treatment. To identify and analyze membrane protein expression, several established techniques have been developed. The most frequently used methods include surface protein biotinylation via a primary amine on a lysine residue or the N-terminus of an exposed protein, followed by proteolysis and affinity enrichment of labeled integral plasma membrane proteins using immobilized beads [9,10], the “shaving” approach consists of digestion of live and intact cells with proteases to generate peptides from the cell surface proteins [7,9]. The peptides generated from both these methods are subsequently analyzed by liquid chromatography-tandem mass spectrometry (LC-MS/MS). Filter-aided sample preparation (FASP) method, which is compatible with the above methods, allows the removal of denaturants, lipids, and nucleic acids prior to trypsin digestion and can act as a ‘proteomic reactor’ for chemical modification, protein digestion, and detergent removal [11,12]. Phenotypic screening approaches, such as hybridoma technology or phage display, also complement the above methods by observing functional changes in cells in response to antibody interactions [13]. These protocols are useful in developing early-stage diagnosis strategies, targeted therapies, and immunotherapies; however, some of these approaches identify peptides based on susceptibility to trypsinization and not immunogenic properties. Hence, a lack of correlation between the abundance of proteins identified and their immunogenicity [4,14]. Another limitation of these approaches is contamination with cytoplasmic proteins, which compromises the specificity of cell surface proteins [9,10]. As a result, surfaceome is rarely used in clinical practice as it is challenging to achieve a precise control of experimental conditions [15].

The “snapshot imprinting” involves the formation of molecularly imprinted nanoparticles (nanoMIPs) at the surface of an intact cell. The selected vinyl functional monomers self-assemble around exposed peptides on cell surface proteins through complementary non-covalent interactions, including hydrogen bonding, electrostatic, and hydrophilic and hydrophobic interactions. Upon initiation, free radical polymerization occurs, forming a stable crosslinked polymeric matrix around the exposed proteins. Following polymerization, the cells are enzymatically digested by trypsin to cleave unprotected cellular proteins. Whereas peptides bound to the nanoMIPs are sterically shielded from proteolysis and are subsequently eluted from the polymeric nanoparticles, concentrated, and sequenced by standard LC-MS/MS, and then analyzed using a bioinformatic tool. Although snapshot imprinting is performed under mild conditions, the presence of radicals may affect cell integrity through oxidative stress. However, this process does not result in complete cell lysis, allowing for the capture of exposed peptides at the precise moment of polymerization. The technique allows for the opportunity for comparative surface proteomic analysis between cancer and normal cells and identifies the molecular markers specific to particular tumors [4,14].

In this study, the “snapshot imprinting” method is used to analyze and characterize the surfaceome of three lung cancer cell lines (A549, H460, NCI-H522) and the BEAS-2B immortalized and non-tumorigenic bronchial epithelial cell line. This selection enables comparison between malignant and non-malignant cells, thereby enabling the identification of cancer-specific molecular markers by nanoMIPs. The innovative use of MIPs offers an alternative approach to surface proteomics in lung cancer and opens new avenues in biomarker discovery.

## 2. Materials and Methods

All reagents used in this project were obtained from Sigma-Aldrich Company Ltd. (Poole, UK), Fisher Scientific Ltd. (Loughborough, UK), or Waters Corporation (Milford, CT, USA) unless otherwise stated.

### 2.1. Cell Culture and Medium

Three non-small cell lung cancer (NSCLC) cell lines were used in this study: A549 (CCL-185), NCI-H460 (HTB-177), and H522 (CRL-5810). One normal human bronchial epithelium BEAS-2B (CRL-3588) cell line was used. All cells were obtained from ATCC. Cells were cultured in T75 culture flasks using Roswell Park Memorial Institute 1640 Medium (RPMI) for A549 and H460, Dulbecco’s Modified Eagle Medium (DMEM) for H522, and LHC-9 Medium (ThermoFisher, Cambridge, UK). DMEM and RPMI were supplemented with 10% Fetal Bovine Serum (FBS) (Sigma-Aldrich, Gillingham, UK) and 1% Penicillin Streptomycin (P/S). LHC-9 Medium was supplemented with 1% P/S. All cells were incubated at 37 °C and under 5% CO_2_ using a Thermo HeraCell 240 incubator. Cells were digested with 0.25% Trypsin–EDTA (Gibco, Thermo Fisher Scientific, Cambridge, UK) and plated for the experiments.

### 2.2. Snapshot Imprinting

Phosphate-buffered saline (PBS, pH 7.4, 10 mM) was prepared using one PBS tablet per 100 mL of HPLC (High-Performance Liquid Chromatography) grade water. This was swirled and sonicated for approximately 10 min. The growth media were carefully decanted from the T75 culture flasks containing each cell line. The cells were washed with PBS (3 × 15 mL), followed by the addition of 15 mL monomeric mixture. The monomeric mixture composition was selected to form a stable, crosslinked polymer network while introducing complementary functional groups capable of forming different non-covalent interactions with amino acid groups on a peptide (Table 1). Monomer ratios are reported as mol percentage (mol%), calculated relative to total molar of monomers and crosslinker. The monomeric mixture (7.05 mM) consisted of N-isopropylacrylamide (NIPAm) (19.5 mg, 0.18 mmol), N, N′-methylene-bis-acrylamide (MBAA) (3 mg, 19.5 µmol), N-tert-butylacrylamide (TBAm) (15 mg, 0.12 mmol) dissolved in 200 µL of ethanol, N-(3-aminopropyl)methacrylamide hydrochloride (3 mg, 16.8 µmol), acrylic acid (1.1 µL, 16.0 µmol) dissolved in 50 mL of PBS. The monomeric mixture was sonicated and degassed under nitrogen for 20 min to minimize oxygen inhibition of the free radical polymerization prior to the addition to the cells. Polymerization was initiated by the addition of 300 µL of the initiation mixture containing potassium persulfate (KPS) (12 mg, 44.4 µmol) and *N*, *N*, *N′*, *N′*-tetramethylethylenediamine (TEMED) (6 µL, 40.3 µmol) dissolved in PBS (400 µL). The polymerization was carried out for 1 h at 20 °C.

### 2.3. Washing and Digestion of the Cell Culture

Each flask was washed with 3 × 15 mL PBS, and the mixture was decanted after each wash. A digestion solution was produced using (for each flask) 3 mg of Trypsin (from porcine pancreas, 1000–2000 BAEE units mg^−1^ solid). It was dissolved in 1 mL of PBS. This was added to the flasks containing 14 mL of the PBS solution and left to digest in an incubator for 72 h at 37 °C.

### 2.4. Separation of the Epitopes of the Cells and nanoMIPs

The solution in the flasks was decanted into 100 kDa centrifuge tubes (Millipore Amicon Ultra-15 Centrifugal Filter, Burlington, MA, USA) and put into the centrifuge (Sigma 3–16 P centrifuge) at 3500 rpm (2355 g) for 10 min to filter through the unreactive functional monomers, cell debris, and leaving the nanoMIP complexes. The nanoMIP complexes in the filter were washed with 4 × 15 mL of HPLC water and centrifuged. After each wash and centrifuge cycle, the cartridge was emptied. HPLC water was heated to ~95 °C. Using a Pasteur pipette, 1 mL of heated water was added to the filter containing the nanoMIPs complexes, then transferred to an Eppendorf tube. The Eppendorf tube was placed into an Eppendorf Thermomixer^®^ (Hamburg, Germany) (C109073 Eppendorf Thermomixer) for 5 min at 800 rpm to disrupt the non-covalent interactions between the imprinted polymers and captured peptides. The solution was transferred back into the filter of the corresponding centrifuge cartridge and centrifuged for 5 min at 3500 rpm (2355 g). The elution step was repeated twice more to maximize peptide recovery. All eluted fractions (3 × 1 mL) were combined and stored at ~−30 °C to preserve the peptide integrity prior to LC-MS/MS analysis.

### 2.5. Transmission Electron Microscopy (TEM) Analysis

TEM imaging was achieved using a JEOL JEM-1400 TEM with an accelerating voltage of 120 kV, equipped with an EMSIS Xarosa digital camera (Münster, Germany) with Radius software (version 2.2). The sample was sonicated for 2 min, and the grids were glow discharged in a Quorum Gloqube plus 30 s @ 30 mA. 5 µL of the sample was added to a freshly glow discharged carbon film grid (400 mesh, AGS160-Agar Scientific Ltd., Rotherham, UK) for 2 min to adsorb. Excess liquid was blotted off, the sample was washed with 2 drops of deionized water, and then left to dry at room temperature before viewing. The statistical analysis of the images was made using open-source software ImageJ version 1.54P.

### 2.6. Scanning Electron Microscopy (SEM) Analysis

SEM images were obtained using a Zeiss Gemini (Oberkochen, Germany) 360 Scanning Electron Microscope with a 5 mm working distance (WD) and an accelerating voltage of 1 kV. 10 µL of the sample was placed directly onto an aluminum stub and left to dry. SEM imaging of the samples utilized the secondary electron detector (SE2) and the inverted Backscattered electron detector (BSE). The images were taken at various magnifications.

### 2.7. Cell Viability Analysis Using CellTiter-Glo (CTG)

Free radical polymerization was initiated using KPS and TEMED, which generate free radicals under mild aqueous conditions. The generated radicals may cause cytotoxicity, therefore affecting cell integrity and protein presentation. cell viability was measured using the CellTiter-Glo Luminescent Cell Viability Assay (Promega, Madison, WI, USA) according to the manufacturer’s instructions to assess the effects of radicals during polymerization. A549, H460, H522, and BEAS-2B were seeded in a 96-well cell culture plate (Corning) at a density of 10,000 cells per 100 µL and incubated for 24 h at 37 °C and 5% CO_2_. After incubation, the cells were washed three times with 100 µL of PBS. For the assay, 100 µL of monomeric mixture was added to each well, and 2 µL of initiator solution was added to the wells designed for polymerization. Plates were incubated at room temperature for 1 h. As a control, cells maintained in culture media were incubated under the same conditions. After incubation, the media and the monomeric mixtures were removed, and the wells were washed three times with 100 µL of PBS. The luminescence signal was measured using a Hidex Sense (Turku, Finland) microplate reader, and viability was calculated relative to the control wells.

### 2.8. Peptide Determination

The samples were left to thaw at room temperature and placed on the SpeedVac (Waltham, MA, USA) for 3 h. The samples were then frozen at −80 °C and lyophilized overnight in the freeze-dryer. The peptides were reconstituted in 25 μL of 0.1% formic acid. Peptide concentrations were measured using the O-phthalaldehyde (OPA) assay. 8 mg of o-Phthalaldehyde (OPA) was added to 100 μL of dimethylformamide (DMF) to form a working solution. A 10 mL solution containing 100 mM boric acid and 1 mg mL^−1^ of Brij was prepared as the assay buffer. 100 μL of the working solution and 2 μL of the 2-mercaptoethanol were added to the 10 mL solution. A 96-well plate was set up for the assay, with 90 μL of water added to the top well, and 50 μL of water added to each of the remaining wells in the column. For the peptide standard, Rho-associated kinase (ROCK) was prepared at a starting concentration of 1 mg mL^−1^. From this stock, 10 μL was added to 90 μL of water in the top well. A doubling serial dilution was performed by transferring 50 μL of solution from one well to the next, mixing thoroughly after each transfer. A blank control well with no peptide was left to account for background. To prepare the wells for the samples, 49 μL, 48 μL, and 47 μL of water were added to separate wells, followed by 1 μL, 2 μL, and 3 μL of the sample to achieve a total volume of 50 μL. Subsequently, 100 μL of the boric acid mixture was added to all wells, and the mixture was incubated at room temperature for 2 min. The final concentrations of the standards were 0 μg mL^−1^, 5 μg mL^−1^, 2.5 μg mL^−1^, 1.25 μg mL^−1^, 0.625 μg mL^−1^, 0.3125 μg mL^−1^, 0.15625 μg mL^−1^, 0.078125 μg mL^−1^. The 96-well plate was read using the GloMax Multi Detection System (Promega). The fluorescence was measured at 490 nm with an excitation wavelength of 340 nm. The concentrations of the samples were calculated by subtracting the background values and correcting them with the standard curve.

### 2.9. LC-MS/MS Procedure

To ensure comparability across all runs, all samples were normalized to a final concentration of 500 ng μL^−1^ by adding a calculated amount of 0.1% formic acid (FA) and 100 fmol yeast alcohol dehydrogenase (ADH) to reconstitute the sample for LC-MS/MS analysis using the calibration line from the OPA assay results. The known amount of ADH protein enabled quantification of the peptides in the sample by comparing peak areas. To optimize LC-MS/MS performance, all samples were injected at a fixed volume of 2 μL with a concentration of 1000 ng μL^−1^.

### 2.10. Nano Ultra-Performance Liquid Chromatography (nanoUPLC)

Sample analysis was performed using Waters nanoACQUITY UPLC (Milford, MA, USA) (Ultra Performance Liquid Chromatography). The samples were initially injected onto a Waters 2G-V/M Symmetry C18 trap column (180 μm × 20 mm, 5 μm) to desalt and remove other impurities and to accurately focus the peptides prior to elution onto the Waters Acquity HSS T3 analytical UPLC column (75 μm × 250 nm,1.8 μm). The solvent flow rate was 0.3 μL/min. Solvent A was LC-MS grade water containing 0.1% formic acid, and solvent B was acetonitrile containing 0.1% formic acid. The following 110 min run time gradient was used: 0 min: 3% B, 30 min: 20% B, 55 min: 42% B, 63 min: 69% B, 65 min: 3% B, 80 min: 3% B, 100 min: 3% B. This was used to maximize peptide separation in the samples, avoiding co-elution and ensuring accurate identification and quantification of proteins and their resulting peptides. For the analytical column, the flow rate was set at 0.3 µL min^−1^, and the temperature was maintained at 40 °C.

### 2.11. Nano Electrospray Ionization Mass Spectrometry

The NanoAcquity UPLC was coupled to a Waters Synapt G2 HDMS mass spectrometer (Waters Corporation, Milford, MA, USA). The Synapt G2-S includes a positive nano electrospray ionization, a StepWave ion guide, a Quadruple, TriWave, and a Time of flight. The mass spectrometer was operated in positive electrospray ionization mode. The capillary voltage was set at 2.4 kV, and the cone voltage was at 30 V. A helium gas flow of 180 mL min−1, and an ion mobility separator (IMS) nitrogen gas flow of 90 mL min^−1^ with a pressure of 2.5 mbar were used. The IMS wave velocity was set at 650 m s^−1^ and the IMS wave height at 40 V. During the acquisition, argon gas was used for collision-induced dissociation (CID). The CID energy was set to 2 V for low energy acquisition, and a 27 to 50 V ramp was applied for high CID energy acquisition. The mass accuracy was maintained throughout the chromatographic run by a [Glu1]-Fibrinopeptide lockspray with a *m*/*z* 785.8427.

### 2.12. Quantification and Statistical Analysis

The data was acquired using MassLynx 4.1. The raw data were analyzed using Progenesis QI for proteomics version 4.2 (Non-Linear Dynamics, Manchester, UK). Progenesis QI enabled the identification of peptides and proteins within samples and quantitative comparison between samples. Progenesis QI also provides quality control metrics to give confidence in experimental conditions, instrument setup, and data analysis. The human UniprotKB (March 2023) database was downloaded and used in FASTA format. The proteomic raw data were searched using strict trypsin cleavage rules with a maximum of two missed cleavages. Cysteine (Carbamidomethyl C) was set as a fixed modification. Deamination N, Oxidation M, and Phosphoryl STY were selected as variable modifications. A minimum of two unique fragments per peptide, a minimum of five fragments per protein, and a minimum of two unique peptides per protein were applied for the parameters of identification. The maximum protein mass was set to 1000 kDa, and the false discovery rate (FDR) for peptide and protein identification was set at a maximum rate of 1%. The Hi-3 relative quantitation method was used, in which the top three most abundant peptides for each protein were employed for protein quantitation. Finally, proteomic data generated with Progenesis QI were exported to Microsoft Excel.

Each experiment was performed in quadruplets, and Microsoft Excel was used to calculate the mean, standard deviation, and fold change. The *p* values were generated in GraphPad Prism 9.0 using the multiple unpaired *t*-test with Welch’s correction for two-group comparisons due to the unequal variance between the groups. Multiple testing correction was applied using the two-stage step-up Benjamini, Krieger, and Yekutieli method to control the FDR at 5%.

### 2.13. Dynamic Lighting Scattering (DLS)

After the elution of the peptides, the corresponding nanoMIPs were characterized using DLS. Samples were re-suspended in HPLC-grade water in a glass vial and sonicated for 5 min (0.99 mL HPLC-grade water, 0.01 mL sample). The solution was transferred to a disposable cuvette, which was placed into the DLS machine for analysis and set at 25 °C. Zetasizer software (version 7.13) was run on Microsoft Windows 10, combined with a Zetasizer Nano range DLS machine (Malvern, UK). The sample was measured for 4 cycles comprising 14 measurements. The size graphs were extracted from the computer software and imported into Microsoft Excel.

### 2.14. Construction of the PPI Network and Identification of Hub Proteins

The Search Tool for the Retrieval of Interacting Genes “http://string-db.org; version 12 (accessed on 4 March 2025)” is a software system commonly used to search for known proteins and predict interactions. The minimum required interaction score was medium confidence (0.4). Cytoscape software (version 3.10.3) was used to construct the PPI network and analyze the hub proteins. The Molecular Complex Detection (MCODE) algorithm, a Cytoscape plugin, was then used to construct the subnetwork and the highly coupled clusters in the PPI network. The criteria for the parameter settings of the MCODE analysis are as follows: degree cut off = 2, node score cutoff  =  0.2, k-core  =  2, and max depth  =  100. Cytoscape plug-in CytoHubba (version 0.1) was used to rank the DEPs in the network using the MCC method.

## 3. Results

### 3.1. Analysis of A549, H460, H522 and BEAS-2B Proteins

**Figure 1:**
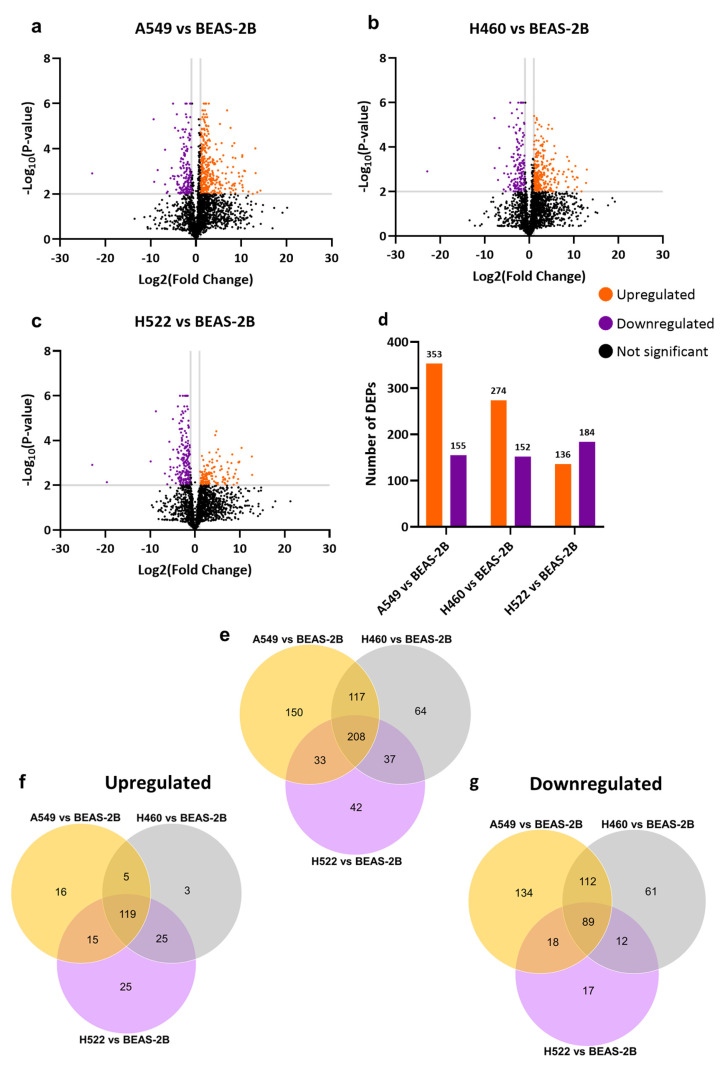
Volcano plots, Histogram, and Venn diagram of protein abundance differences in A549, H460, H522 vs. BEAS-2B following snapshot imprinting. (**a**–**c**) Volcano plots showing differential protein abundance in A549, H460, and H522 lung cancer cells compared to BEAS-2B cells (n = 4 biological replicates). Each point represents an individual protein. Orange points indicate significantly upregulated proteins, and purple points indicate significantly downregulated proteins, defined by an FC ≥ 2 and ≤ 0.5, with *p*-value ≤ 0.01. Black spots indicate proteins that did not meet significance criteria. Vertical gray lines indicate the FC thresholds, while the horizontal gray line represents the *p*-value threshold. For clarity, *p*-values of <1.00 × 10^−6^ were set at 1.00 × 10^−6^ to allow inclusion of significant proteins in the plot. (**d**) The bar graph summarizes the numbers of upregulated and downregulated proteins identified in each comparison based on the defined thresholds. (**e**) The Venn diagram illustrates the overlap between the total number of DEPs in A549 vs. BEAS-2B, H460 vs. BEAS-2B, and H522 vs. BEAS-2B. (**f**) Venn diagram showing the upregulated DEPs and (**g**) downregulated DEPs identified in the three comparisons.

Snapshot imprinting was used to compare the A549, H460, and H522 cell lines with BEAS-2B, using label-free quantitative proteomics to understand the differences. The proteomics analysis identified 2381 proteins (Table S1). The fold change and *p*-value for each protein were calculated (Table S2). A fold change (FC) of ≥2 and ≤0.5 was applied to identify and prioritize proteins that exhibit robust and biologically meaningful changes in abundance between two different lung cancer cell lines, and a *p*-value ≤ 0.01 was used to assess the statistical significance to identify differentially expressed proteins (DEPs). Volcano plots and a histogram were used to visualize the significant DEPs between the cell lines and to categorize the up- and downregulated proteins (Figure 1a,b). The comparison between A549 and BEAS-2B cell lines, a total of 508 DEPs were identified. Of these, 353 proteins were upregulated, and 115 were downregulated. 426 DEPs were identified when H460 was compared to BEAS-2B, of which 274 proteins were upregulated, and 152 were downregulated. Additionally, the comparison between H522 and BEAS-2B identified 320 DEPs, including 136 upregulated proteins and 184 down-regulated proteins. The top 10 upregulated and downregulated DEPs in A549, H460, and H522, with their UniProt Gene Ontology subcellular location, are listed in (Tables S3-1–S3-6). It can be noted that 45% of the top 10 upregulated and downregulated proteins are intracellular, while 50% are multi-compartmental according to the UniProt GO annotations. This reflects the highly abundant intracellular proteins like the cytoskeleton and ribosomes, and the dynamic trafficking of proteins, often moving between intracellular compartments and the extracellular environment. To reduce interference from highly abundant proteins and identify biologically relevant proteins, the overlapping DEPs across all cancer cell lines relative to BEAS-2B were analyzed. The DEPs in each group (A549 vs. BEAS-2B, H460 vs. BEAS-2B, and H522 vs. BEAS-2B) were compared; 208 proteins were found to overlap, with 89 DEPs being upregulated and 119 being downregulated (Figure 1e–g). These proteins were further investigated to identify potential NSCLC targets, as they are likely to represent biologically relevant lung cancer-associated proteins captured by molecular imprinting. By focusing on these proteins, it minimizes the random or cell-line-specific variations.

### 3.2. Functional Enrichment Analysis

**Figure 2:**
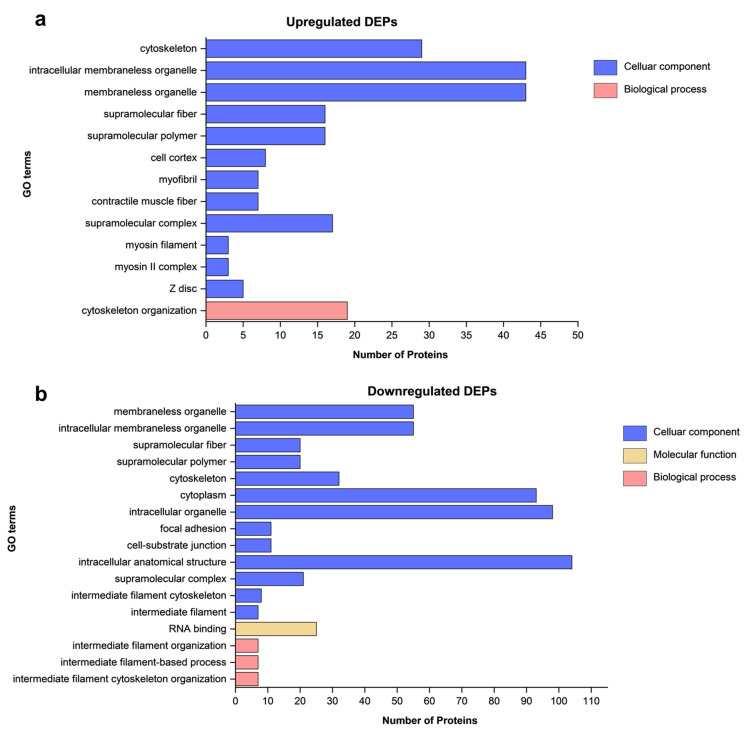
Functional enrichment analysis of DEPs. (**a**) GO enrichment analysis of the upregulated and (**b**) downregulated DEPs based on cellular component, biological process, and molecular function.

Gene ontology (GO) was used to perform functional analysis of overlapping DEPs, which were separated into two graphs: upregulated and downregulated. The DEPs were categorized into three functional groups: biological process (BP), molecular function (MF), and cellular component (CC). The GO analysis results (Figure 2) show the enriched GO terms for the 89 upregulated and 117 out of 119 downregulated DEPs, sorted by *p*-value, using a significance threshold of <0.05. ABCA11P (Q4W5N1) was made obsolete, and HLA-A (P13746-2, P30443) was merged into HLA-A (P04439). The up-regulated DEPs were primarily located in the cytoskeleton and the intracellular membraneless organelle (CC) and were associated with cytoskeleton organization (BP). The down-regulated DEPs were mainly enriched in the membraneless organelle, intracellular membraneless organelle (CC), and associated with intermediate filament-based process, intermediate filament organization (BP), and RNA binding (MF).

To provide an overview of the subcellular localization of the 206 overlapping DEPs captured by snapshot imprinting, GO-CC terms were used. The analysis included both statistically significant (FDR < 0.05) and non-significant (FDR > 0.05) results. The proteins were classified into extracellular organelle, cell surface, plasma membrane, and intracellular categories. The percentage contributions of each category and the FDR value were summarized in Table 2, enabling the comparison between surface-associated proteins and intracellular proteins captured by the nanoMIPs.

### 3.3. Protein–Protein Interaction PPI Network Analysis

**Figure 3:**
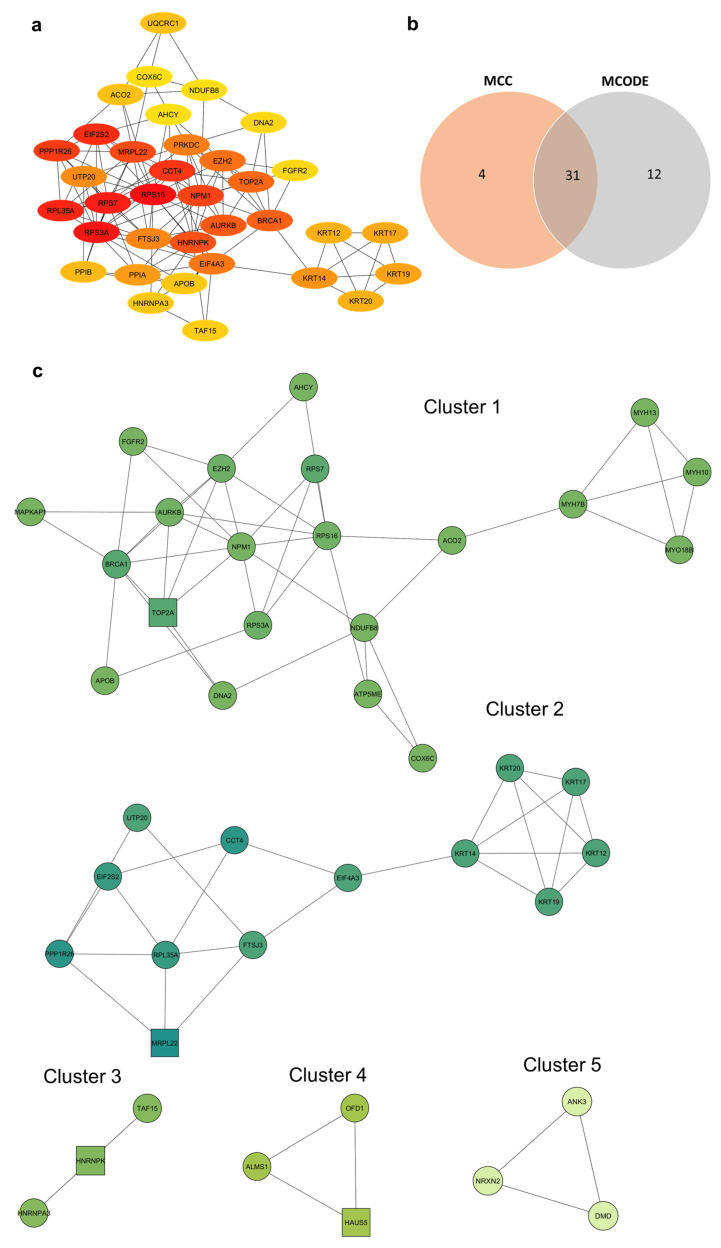
Identification of hub proteins using PPI analysis. (**a**) The top 35 hub proteins were ranked using the MCC algorithm. Node color intensity reflects ranking, with the dark red color indicating top-ranked proteins, while the pale-yellow color indicates low-ranked proteins. (**b**) Venn diagram showing the overlap between proteins identified as hubs by MCC ranking and those clustered by MCODE. (**c**) MCODE analysis identifies densely connected protein clusters (MCODE 1–5) within the PPI network that represent stable functional units within the cell.

The 208 overlapping DEPs were input into the Cytoscape 3.10.3 software using the STRING tool 9 [16,17]. The Cytoscape plugin cytoHubba ranks DEPs using the Maximal Clique Centrality (MCC) algorithm [18]. In total, 205 out of 208 proteins were included in the PPI analysis, as three proteins were merged and marked as obsolete (Table S4). The MCC approach was found to be the most accurate at predicting important proteins known as “hub proteins” in the PPI network. Proteins with high MCC scores are considered “hub proteins” because they interact with many other proteins (i.e., form cliques) within the network. The top 35 MCC-ranked proteins with a score of ≥10 from the 205 DEPs PP1 network were selected (Figure 3a). The MCODE plugin was also used to identify hub proteins within the PPI network by detecting densely connected functional clusters of the 205 DEPs [19]. The DEPs were screened with a node score cutoff of 0.2, k-core of 2, and a degree cutoff of 2. Five clusters were identified using MCODE; cluster one had a score of 4.4 and contained 21 DEPs. The other 4 clusters had scores of 4, 3, 3, and 3, respectively (Figure 3b). The square nodes within the cluster represent the most highly connected and central protein to the cluster’s structure and function. The top 35 MCC DEPs were compared to the DEPs identified in the MCODE clusters. A total of 31 DEPs (Table S5) overlapped between these two analyses (Figure 3c). This comparison aimed to identify biologically significant “hub” proteins from the highly interconnected hub proteins (MCC) and densely clustered functional groups (MODE), as these densely connected regions are less likely to occur by chance.

### 3.4. Identification of Potential NSCLC Protein Targets

To identify potential lung cancer targets from the hub proteins and assess their biological significance, the protein list was cross-referenced with the existing literature and the protein expression data found in the Human Protein Atlas (HPA) (http://www.proteinatlas.org/ (accessed on 17 March 2025), which incorporates mass spectrometry-based proteomics data from the Clinical Proteomic Tumor Analysis Consortium (CPTAC) and immunohistochemistry (IHC) staining of lung adenocarcinoma (LUAD) and lung squamous cell carcinoma (LUSC) tissue and normal lung tissue. Five proteins (NPM1, TOP2A, EZH2, PRKDC, and HNRNPK) were identified as potential targets for diagnostic and therapeutic applications. These proteins are summarized in Table 3 along with their average amounts, fold changes, and *p*-values. A fold change of ≥2 indicates upregulation, while ≤0.5 signifies downregulation.

### 3.5. Selectivity

**Figure 4:**
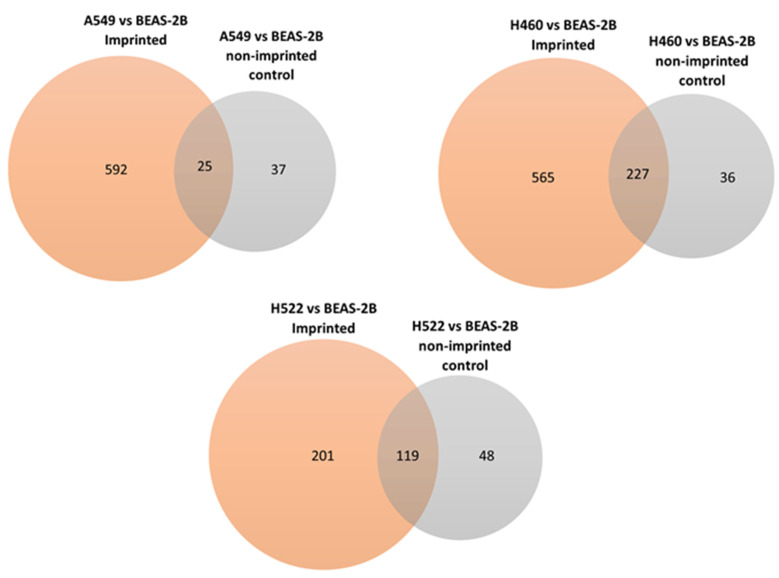
Comparison between DEPs identified from snapshot imprinting and non-imprinted control.

As a control experiment to assess the selectivity of protein capture via molecular imprinting, the A549, H460, H522, and BEAS-2B cell lines underwent a snapshot imprinting protocol without the addition of the polymerization initiator. Therefore, the proteins on the cell surface were digested without the protection of nanoMIPs. This experiment mimics the “shaving” approach used in surface protein analysis, followed by LC-MS/MS analysis. A total of 508, 426, and 320 DEPs were identified when A549, H460, and H522 cell lines, respectively, were compared to BEAS-2B using snapshot imprinting (Figure 4). Whereas, 295, 263, and 167 DEPs were identified when A549, H460, and H522 cell lines, respectively, were compared to BEAS-2B for the non-imprinting control. Across all lung cancer cell lines, when compared to BEAS-2B, the imprinted polymers consistently captured more DEPs, including unique DEPs that were not detected in the non-imprinted control condition. The proteins that are detected in both the imprinting and non-imprinting control or just the non-imprinting control probably represent highly abundant cytosolic and extracellular proteins that can adsorb onto the filter membrane in the centrifuge cartridge during the wash steps or are associated with proteins that were captured by partially or spontaneously self-polymerized monomers through non-covalent interactions during the 1 h period.

### 3.6. Cell Viability

**Figure 5:**
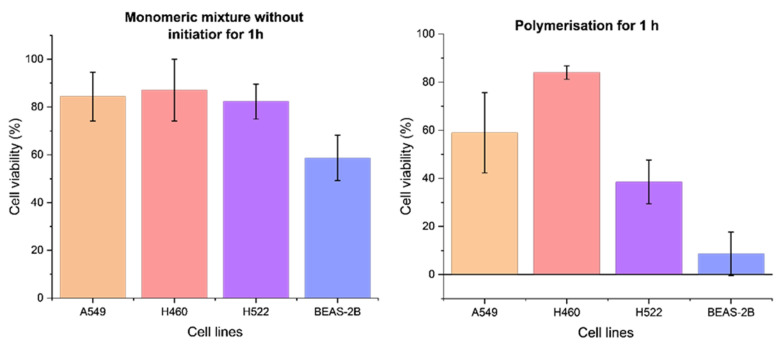
CTG assay data showing the effects of the monomeric mixture and polymerization process after 1 h of exposure. Data represent ± SD of four biological replicates.

The effects of polymerization and the monomeric mixture on cell viability were assessed by using the CellTiter-Glo (CTG) luminescent assay (Figure 5). The two conditions were compared: cells exposed to the monomeric mixture for 1 h at room temperature and cells subjected to polymerization for 1 h at RT. The average percentages in non-polymerizing conditions ranged from 82 to 87% for the lung cancer cell lines and 59% for BEAS-2B cells. Upon initiation of polymerization, cell viability decreased to (39–84%) for lung cancer cell lines and 9% for BEAS-2B. These results confirm that the individual monomers are largely biocompatible with cancer cells and are non-toxic to cultured cells. However, the polymerization process led to decreased viability, particularly in H522 and BEAS-2B cells. The reduction in viability can be attributed to the polymerization occurring in non-optimal conditions (room temperature, 1 h), which deviate from the optimum conditions for incubating (37 °C, 5% CO_2_), the absence of culture medium, and the presence of radicals generated by potassium persulfate and *N*, *N*, *N’*, *N’*-tetramethylethylenediamine. The magnitude of the effects varied across all cell types, suggesting possible differences related to cell morphology, membrane composition, and tolerance to chemical stress that may lead to cell death. The observed reduction in viability supports the interpretation that a portion of cells likely undergo cell death via necrosis or apoptosis.

### 3.7. Characterization of Eluted nanoMIPs

**Figure 6:**
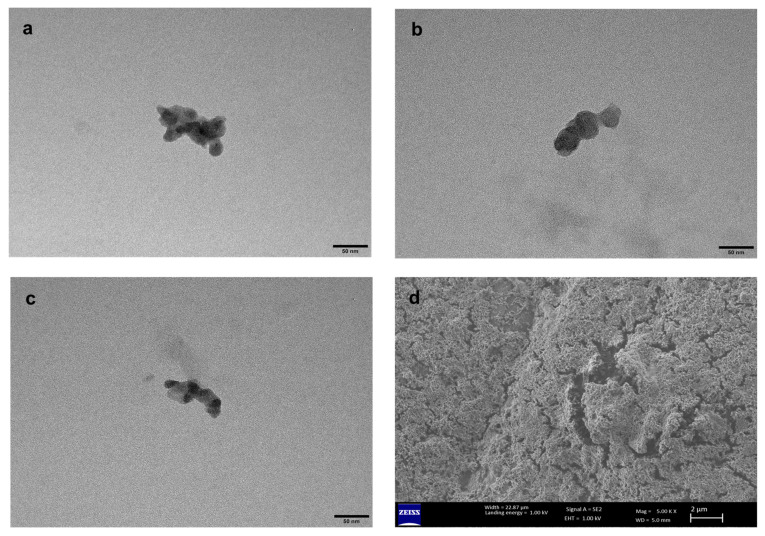
(**a**–**c**) TEM and (**d**) SEM images of eluted nanoMIPs.

The DLS average hydrodynamic diameter was 213 nm ± 14.9, with a Polydispersity index of 0.305 nm ± 0.019 in water. The TEM images of nanoMIPs (Figure 6a–c) had an average particle size of 111 nm ±13.0 measured using ImageJ. The SEM (Figure 6d) shows the surface topology of a highly crosslinked, heterogeneous surface structure with visible pores. The discrepancy between DLS and TEM sizes reflects the differences in each technique: DLS measures the hydrodynamic diameter of nanoparticles in suspension, which includes the surface coating and the surrounding layer of solvent molecules that move with the particle under Brownian motion in a solution, whereas TEM measures the physical core size of the dried particles under vacuum.

## 4. Discussion

The snapshot imprinting technique has been used to capture and analyze the cell surface proteome of lung cancer cells at a precise moment in time. The monomeric mixture combined structural, crosslinking, and functional monomers to form a responsive polymer network: NIPAm and TBAm formed the main backbone and tuned thermoresponsiveness and hydrophobicity; MBAA provided covalent crosslinking; APMA introduced positively charged amine functionality; and acrylic acid supplied negatively charged carboxyl groups. The diversity of monomer functional groups enabled complementary interactions with a broad spectrum of protein side chains [20]. The chemical rationale was so the polymer could form highly crosslinked polymers and functional groups could form non-covalent interactions with the peptides, while limiting the cytotoxic effects. While the monomeric mixture was tolerated by the different lung cancer cells, the free radical polymerization process generated reactive radical species, resulting in oxidative stress, plasma membrane dysfunction, and cell death. Although repeated wash steps removed the majority of detached cells and soluble intracellular proteins, proteins from the compromised adhered cells may be captured during molecular imprinting. As a result, the types and numbers of proteins identified differ between experiments, depending on the proportions of viable and compromised cells available. Therefore, identifying common DEPS across cell lines causes fewer discrepancies.

The 206 overlapping DEPs GO cellular component analysis revealed that 83% (FDR < 0.05) of the shared DEPs were intracellular organelles, while 30% were associated with the plasma membrane, and 17% were extracellular organelles but did not reach statistical significance. The overall percentage exceeding 100% reflects the fact that these proteins reside to multiple cellular compartments, indicating dynamic trafficking rather than just intracellular confinement. The presence of intracellular proteins has been observed in classical surface approaches, but is not entirely understood. To explain the phenomenon authors have presented the following possibilities: (1) cytoplasmic and nuclear proteins come from cell lysis (necrosis) and apoptosis, thus contaminating the “surfaceome” fraction [21,22]; (2) cytoplasmic, mitochondrial and even nuclear proteins have reached the surface by exporting/secretory machinery through canonical and non-canonical secretion pathways [23,24], the integration of extracellular vesicles [25,26], and adsorption of proteins released during cell death or mitosis [4,14]. This suggests that the detection of a large number of intracellular proteins in snapshot imprinting is a combination of sensitivity to the polymerization process and the preferential capture of highly abundant, multi-compartmental proteins.

The GO biological process of the shared DEPs included cytoskeleton organization and intermediate filament-based processes. These pathways play a key role in the maintenance of cell structure, cell motility, division, and intracellular processes; however, their deregulation in cancer is known to promote tumor progression, malignancy, invasiveness, and metastatic behavior [27,28]. Because these biological processes reflect the highly interconnected molecular network, changes in individual proteins may not fully reflect their biological significance. Therefore, PPI analysis was performed using the MCC and MCODE algorithms to identify individual highly connected hub proteins that may act as central regulators of the affected NSCLC pathways, therefore reducing bias from protein abundance or subcellular localization.

The hub proteins were cross-referenced with HPA data, specifically focusing on CPTAC protein expression data and IHC staining profiles. This led to the identification of five proteins (NPM1, TOP2A, EZH2, PRKDC, HNRNPK) that were significant in both LUAD and LUSC tissue compared to normal lung cancer tissue. NPM1 is a molecular chaperone involved in nucleocytoplasmic transportation of nucleic acids and proteins, and cell cycle regulation. It has been reported to be upregulated in LUAD, where its expression is associated with poor progression, cell proliferation, and invasion via the EGFR/MAPK pathway [29]. TOP2A is a nuclear enzyme that plays an essential role in DNA replication, transcription, and recombination [30,31]. It is frequently overexpressed in NSCLC, where elevated levels correlate with tumor metastasis, progression, and worse prognosis in NSCLC patients [32,33]. EZH2 is a histone methyltransferase that performs epigenetic functions [34]. EZH2 signaling promotes proliferation, metastasis, and its dysregulation in NSCLC is associated with poor prognosis and worse overall survival, supporting its role as a prognostic marker [35,36,37]. EZH2 inhibitors have investigated in preclinical models and clinical trials, primarily in combination with other therapies to improve patient outcome [38,39]. PRKDC, also known as DNA-PKcs, plays a critical role in the DNA double-strand break repair and recombination [40,41,42]. Its overexpression in NSCLC patients was linked to poor prognosis and resistance to chemotherapy [43,44]. This highlights its potential as both a prognostic and predictive biomarker. [41,43,44]. DNA-PKcs has also been explored as a therapeutic target in both NSCLC solid tumors and cell lines using DNA-PK inhibitors such as peposertib (formally M3814) and AZD7648 [45,46,47]. hnRNPK is an RNA- and DNA-binding component of the ribonucleoprotein in both transcriptional and post-transcriptional mechanisms, and its expression has been correlated with cell migration, invasion, and metastasis in lung cancer [48,49,50]. The detection of these hub proteins by snapshot imprinting highlights how nanoMIPs can capture diagnostically and prognostically meaningful proteins in lung cancer cells and emerging therapeutic targets.

Although this approach successfully enriched several emerging lung cancer protein targets, it cannot replace other protocols used in surfaceome research, as the non-optimal conditions influence the types of proteins captured. Additionally, the use of lung cancer cell lines does not accurately reflect tumor heterogeneity. While the findings demonstrate proof of concept, future studies should be conducted on clinically relevant liquid biopsies, such as pleural effusions and bronchoalveolar lavage fluids, as the next step towards incorporating the nanoMIP-based proteomics pipeline to enable minimally invasive detection of cancer-associated proteins.

In conclusion, this study demonstrates the successful application of molecularly imprinted polymer nanoparticles for snapshot imprinting of lung cell surfaces, enabling the identification of differentially expressed proteins across different lung cancer cell lines and non-cancerous control cells. While some expression trends observed in this study may differ from other proteomics studies, this discrepancy reflects the nature of the snapshot imprinting method. Therefore, the aim of this work was not to replicate known differential expression patterns but to demonstrate that nanoMIPs can selectively capture biologically relevant proteins.

## Figures and Tables

**Table 1:** Chemical composition of the monomeric mixture used in snapshot imprinting.

Component	Function	mol%
NIPAM	Backbone	55.1
TBAM	Hydrophobic	34.1
APMA	Cationic	4.8
Acrylic acid	Anionic	4.5
MBAA	Crosslinker	5.5
Total	-	100

**Table 2:** GO cellular component classification of overlapping DEPs.

GO-CC Terms	Number of Proteins	Percentage (%)	FDR
Extracellular organelle	34	17	1.81 × 10^−1^
Cell surface	11	5	1.00 × 10^0^
Plasma membrane	62	30	1.00 × 10^0^
Cytosol	79	39	2.91 × 10^−2^
Cytoplasm	156	76	6.00 × 10^−4^
Intracellular organelle	172	83	3.07 × 10^−6^

**Table 3:** Potential NSCLC protein targets present in A549, H460, and H522 cell lines with significant fold change and *p*-value. STD: Standard deviation.

Protein Name	Gene Name	A549		H460		H522		BEAS-2B		A549 vs. BEAS-2B		H460 vs. BEAS-2B		H522 vs. BEAS-2B	
		Average Amount (fmol)	STD	Average Amount (fmol)	STD	Average Amount (fmol)	STD	Average Amount (fmol)	STD	Fold Change	*p*-Value	Fold Change	*p*-Value	Fold Change	*p*-Value
Nucleophosmin	NPM1	19.72	4.66	17.18	5.62	15.29	8.09	3.14	0.59	6.28	1.70 × 10^−5^	5.47	1.87 × 10^−4^	4.87	3.77 × 10^−3^
DNA topoisomerase 2-alpha	TOP2A	9.66	5.21	15.04	6.10	10.11	6.10	1.96	0.48	4.92	4.06 × 10^−3^	7.66	4.94 × 10^−4^	5.15	6.85 × 10^−3^
Histone-lysine N-methyltransferase	EZH2	13.85	10.33	18.43	16.87	10.09	8.58	45.19	10.54	0.31	3.20 × 10^−5^	0.41	2.61 × 10^−3^	0.22	5.00 × 10^−6^
DNA-dependent protein kinase catalytic subunit	PRKDC	10.65	3.05	9.44	5.27	8.69	4.65	65.11	21.31	0.16	1.52 × 10^−4^	0.14	1.04 × 10^−4^	0.13	1.03 × 10^−4^
Heterogeneous nuclear ribonucleoprotein K	HNRNPK	5.68	2.81	5.09	3.49	4.29	2.82	28.38	5.39	0.20	1.00 × 10^−6^	0.18	1.00 × 10^−6^	0.15	1.00 × 10^−6^

## Data Availability

The original contributions presented in this study are included in the article/Supplementary Material. Further inquiries can be directed to the corresponding author.

## Supplementary Materials

The following supporting information can be downloaded at https://www.mdpi.com/article/10.3390/polym18020281/s1, Table S1: Normalized data; Table S2: Statistical analysis; Tables S3-1–S3-6: Top10 up-downregulated DEPs; Table S4: 208 overlapping DEPs PPI network; Table S5: 31 hub proteins.

## Abbreviations

The following abbreviations are used in this manuscript:

nanoMIPs	Molecularly imprinted polymer nanoparticles
DEPs	Differentially expressed proteins
LC-MS/MS	Liquid chromatography-tandem mass spectrometry
FASP	Filter-aided sample preparation
NSCLC	non-small lung carcinoma
IMS	ion mobility separator
CID	collision-induced dissociation
FDR	False discovery rate
GO	Gene ontology
BP	Biological process
MF	Molecular function
CC	Cellular component
PPI	Protein–protein interaction
MCC	Maximal Clique Centrality
HPA	Human Protein Atlas
CPTAC	Clinical Proteomic Tumor Analysis Consortium
LUAD	lung adenocarcinoma
NPM1	Nucleophosmin
TOP2A	DNA topoisomerase 2-alpha
EZH2	Histone-lysine N-methyltransferase
ANXA3	Annexin A3
PRKDC	DNA-dependent protein kinase catalytic subunit
HNRNPK	Heterogeneous nuclear ribonucleoprotein K
LRPPRC	Leucine-rich PPR motif-containing protein—mitochondrial
