# Supplementary material for: Molecularly Imprinted Polymer Nanoparticles for Lung-Cancer-Cell-Surface Proteomics
Source: Polymers (Basel). 2026 Jan 20;18(2):281. doi: 10.3390/polym18020281 (PMC12845741; doi:10.3390/polym18020281)
Supplement: Supplementary file 1 [file polymers-18-00281-s001.zip › Table S5-31 hub proteins.pdf]

Accession	Name	Gene Name	MCC Score	Rank	MCODE score	Rank	Subcellular location
P62249	Small ribosomal subunit protein uS9	RPS16	647	1	16	11	Cytoplasm, Nucleus, Nucleolus
P61247	Small ribosomal subunit protein eS1	RPS3A	591	2	7	13	Cytoplasm, Nucleus, Nucleolus
P62081	Small ribosomal subunit protein eS7	RPS7	564	3	6	15	Cytoplasm, cytoskeleton, Centrosome, Nucleus, Nucleolus
P18077	Large ribosomal subunit protein eL33	RPL35 A	386	4	8	12	Cytoplasm
P20042	Eukaryotic translation initiation factor 2 subunit 2	EIF2S2	366	5	4	22	Cytoplasm, cytosol
P50991	T-complex protein 1 subunit delta	CCT4	296	6	3	25	Cell projection, Cytoplasm, centrosome, cilium basal body, cytoskeleton
Q5T8A7	Protein phosphatase 1 regulatory subunit 26	PPP1R2 6	272	7	5	20	Nucleus, Nucleolus
P06748	Nucleophosmi n	NPM1	260	8	43	1	Cytoplasm, cytoskeleton, centrosome, Nucleus, nucleolus, nucleoplasm
Q9NWU5	Large ribosomal subunit protein uL22m	MRPL2 2	153	9	4	22	Mitochondrio n
P61978	Heterogeneous nuclear ribonucleoprot ein K	HNRNP K	117	10	2	30	Cytoplasm, cell projection, podosome, Nucleus, nucleoplasm
Q96GD4	Aurora kinase B	AURKB	99	11	32	4	Nucleus, Chromosome, centromere,

							cytoplasm, cytoskeleton, spindle, midbody
P38398	Breast cancer type 1 susceptibility protein	BRCA1	81	12	35	3	Nucleus, Chromosome, Cytoplasm
P11388	DNA topoisomerase 2-alpha	TOP2A	65	13	26	5	Cytoplasm, Nucleus, Nucleolus, nucleoplasm
Q15910	Histone-lysine N-methyltransferase EZH2	EZH2	63	14	38	2	Nucleus
P38919	Eukaryotic initiation factor 4A-III	EIF4A3	56	15	3	25	Cytoplasm, Nucleus, Nucleus speckle
Q8IY81	pre-rRNA 2'-O-ribose RNA methyltransferase FTSJ3	FTSJ3	45	17	4	22	Nucleus, nucleolus
O75691	Small subunit processome component 20 homolog	UTP20	38	18	2	30	Nucleus, nucleolus
P02533	Keratin, type I cytoskeletal 14	KRT14	31	19	25	6	Cytoplasm, Nucleus
P08727	Keratin, type I cytoskeletal 19	KRT19	29	21	24	7	Plasma membrane, costamere, cytoskeleton, cytosol, Intermediate filament, extracellular exosome
Q04695	Keratin, type I cytoskeletal 17	KRT17	26	22	24	7	Cytoplasm
Q99456	Keratin, type I cytoskeletal 12	KRT12	24	23	24	7	Cytoskeleton, cytosol, extracellular exosome
P35900	Keratin, type I cytoskeletal 20	KRT20	24	23	24	7	Cytoplasm
Q99798	Aconitate hydratase, mitochondrial	ACO2	15	26	3	25	Mitochondrion
P51991	Heterogeneous nuclear ribonucleoprotein A3	HNRNPA3	14	28	2	30	Nucleus

P04114	Apolipoprotein B-100	APOB	14	28	2	30	Cytoplasm, Secreted, Lipid droplet
Q92804	TATA-binding protein-associated factor 2N	TAF15	12	30	2	30	Nucleus, Cytoplasm
P21802	Fibroblast growth factor receptor 2	FGFR2	11	31	6	15	Cell membrane, Golgi apparatus, Cytoplasmic vesicle
P51530	DNA replication ATP-dependent helicase/nuclease DNA2	DNA2	11	31	3	25	Nucleus, Mitochondrion
P09669	Cytochrome c oxidase subunit 6C	COX6C	10	33	2	30	Mitochondrion inner membrane
O95169	NADH dehydrogenase [ubiquinone] 1 beta subcomplex subunit 8, mitochondrial	NDUFB8	10	33	5	20	Mitochondrion inner membrane
P23526	Adenosylhomocysteinase	AHCY	10	33	2	30	Cytoplasm, Nucleus, Endoplasmic reticulum
