# Supplementary material for: Molecularly Imprinted Polymer Nanoparticles for Lung-Cancer-Cell-Surface Proteomics
Source: Polymers (Basel). 2026 Jan 20;18(2):281. doi: 10.3390/polym18020281 (PMC12845741; doi:10.3390/polym18020281)
Supplement: Supplementary file 1 [file polymers-18-00281-s001.zip › Tables S3-1-S3-6-Top 10 Up-Downregulated DEPs.pdf]

Table S3-1. Top 10 upregulated DEPs through snapshot imprinting analysis comparing A549 to BEAS-2B cell line. STD: Standard deviation

Gene Name	Protein Name	A549 Average Amount (fmol)	STD	BEAS-2B Average Amount (fmol)	STD	Fold Change	P-value	Location*
SH3GL1	Endophilin-A2	9.66	1.30	1.35	0.52	7.17	< 1.00E-06	Cytoplasm, podosome
SPIRE1	Protein spire homolog 1	17.82	2.77	3.73	1.94	4.78	< 1.00E-06	Cytoskeleton, plasma membrane, perinuclear region, mitochondrial outer membrane
PCNT	Pericentrin	22.82	1.99	4.87	0.80	4.68	< 1.00E-06	Centrosome, centriolar satellite membrane
MRPL22	39S ribosomal protein L22_ mitochondrial	68.06	5.34	17.37	9.99	3.92	< 1.00E-06	Mitochondrion
CABP5	Calcium-binding protein 5	29.26	4.87	8.88	2.25	3.29	< 1.00E-06	Cytoplasm, cytosol
THAP11	THAP domain-containing protein 11	21.18	4.23	0.18	0.24	119.13	2.00E-06	Nucleus, cytoplasm
KRT1	Keratin_ type II cytoskeletal 1	9.61	2.00	2.39	1.74	4.03	2.00E-06	Plasma membrane, keratin filament, cytoplasm, cytoskeleton, extracellular space, extracellular exosome, nucleus
ZNF394	Zinc finger protein 394	1.83	0.34	0.62	0.25	2.92	2.00E-06	Nucleus
PRPS2	Ribose-phosphate pyrophosphokinase 2	12.08	2.13	4.41	1.37	2.74	2.00E-06	Cytoplasm, cytosol
XDH	Xanthine dehydrogenase/oxidase	0.31	0.07	0.06	0.04	4.93	3.00E-06	Cytoplasm, cytosol, extracellular space, peroxisome

*The locations for each protein are based on both UniprotKB and GO annotations.

Table S3-2. Top 10 downregulated DEPs through snapshot imprinting analysis comparing A549 to BEAS-2B cell line

Gene Name	Protein Name	A549 Average Amount (fmol)	STD	BEAS-2B Average Amount (fmol)	STD	Fold Change	P value	Location
ANXA6	Annexin A6	3.62	0.90	15.11	2.56	0.24	1.00E-06	Cytoplasm, extracellular exosome, focal adhesion, plasma membrane, nucleus, lysosomal membrane, late endosome membrane
KRT17	Keratin_ type I cytoskeletal 17	12.59	2.81	27.05	3.60	0.47	<1.00E-06	Cytoplasm, cytoskeleton
MTAP	S-methyl-5'-thioadenosine phosphorylase	7.21	2.01	17.35	2.03	0.42	<1.00E-06	Cytoplasm, nucleus, extracellular exosome
HNRNPK	Heterogeneous nuclear ribonucleoprotein K	5.68	2.81	28.38	5.39	0.20	<1.00E-06	Cytoplasm, nucleus, podosome, extracellular exosome, membrane, focal adhesion, chromatin
KRT20	Keratin_ type I cytoskeletal 20	0.21	0.22	6.93	1.24	0.03	<1.00E-06	Cytoplasm, cytoskeleton, intermediate filament cytoskeleton
PPP1R12B	Protein phosphatase 1 regulatory subunit 12B	1.37	0.68	6.33	1.37	0.22	3.00E-06	Cytoskeleton, stress fiber
RPRD2	Regulation of nuclear pre-mRNA domain-containing protein 2	1.09	0.38	20.31	4.11	0.05	3.00E-06	nucleoplasm, transcription perinitiation complex
VPS13C	Vacuolar protein sorting-associated protein 13C	19.51	7.71	46.85	7.40	0.42	4.00E-06	Cytoplasm, cytosol, extracellular exosome, Mitochondrion outer membrane, Lipid droplet, endoplasmic reticulum membrane, lysosome membrane, late endosome membrane
NKX2-6	Homeobox protein Nkx-2.6	0.39	0.19	1.65	0.36	0.24	4.00E-06	Nucleus
CD151	CD151 antigen	0.01	0.01	8.12	1.82	0.002	5.00E-06	Cell membrane, basement membrane, cell surface, focal adhesion, cytosol

Table S3-3. Top 10 upregulated DEPs through snapshot imprinting analysis comparing H460 to BEAS-2B cell line.

Gene Name	Protein name	H460 Average Amount (fmol)	STD	BEAS-2B Average Amount (fmol)	STD	Fold Change	P value	Location
PHLDB2	Pleckstrin homology-like domain family B member 2	60.61	9.47	28.72	6.59	2.11	4.00E-06	Cytoplasm, cell cortex, plasma membrane, podosome, intermediate filament cytoskeleton
PLEKHA5	Pleckstrin homology domain-containing family A member 5	12.89	2.61	4.14	2.09	3.11	5.00E-06	Cytoplasm, membrane, nucleoplasm
MRPL22	39S ribosomal protein L22_ mitochondrial	56.79	11.95	17.37	9.99	3.27	6.00E-06	Mitochondrion
DMD	Dystrophin	24.83	4.63	9.54	4.32	2.60	8.00E-06	Cell membrane, cytoskeleton, cytosol, filopodium, filopodium membrane
PCDP1	Primary ciliary dyskinesia protein 1	63.59	15.47	3.13	1.36	20.30	1.00E-05	Cilium axoneme, cytoskeleton, cytoplasm, extracellular region
EXOC7	Exocyst complex component 7	13.41	3.19	2.84	1.26	4.72	1.00E-05	cytosol, cell membrane, midbody ring, plasma membrane, centriolar satellite
CCDC144A	Coiled-coil domain-containing protein 144A	71.62	18.28	11.01	6.66	6.51	1.20E-05	Unknown
SUCLG2	Succinyl-CoA ligase [GDP-forming] subunit beta_ mitochondrial	1.33	0.35	0.04	0.05	34.43	1.50E-05	Mitochondrion, plasma membrane
TJAP1	Tight junction-associated protein 1	684.63	177.42	37.25	18.86	18.38	1.50E-05	trans-Golgi network, tight junction, cell membrane
APOB	Apolipoprotein B-100	43.79	9.91	12.26	3.61	3.57	1.60E-05	Cytoplasm, extracellular space, extracellular exosome, lipid droplet, endoplasmic reticulum, endosome lumen, lysosomal lumen

Table S3-4. Top 10 downregulated DEPs through snapshot imprinting analysis comparing H460 to BEAS-2B cell line.

Gene Name	Protein name	H460 Average Amount (fmol)	STD	BEAS-2B Average Amount (fmol)	STD	Fold Change	P value	Location
KRT17	Keratin_ type I cytoskeletal 17	10.72	2.10	27.05	3.60	0.40	<1.00E-06	Cytoplasm, cytosol, cytoskeleton
MTAP	S-methyl-5' thioadenosine phosphorylase	5.61	2.91	17.35	2.03	0.32	<1.00E-06	Cytoplasm, Nucleus, extracellular exosome
ALMS1	Alstrom syndrome protein 1	9.46	3.31	31.71	5.44	0.30	<1.00E-06	Cytoplasm, centrosome, cilium basal body, spindle pole, mitochondrion, nucleoplasm
ANXA6	Annexin A6	3.93	1.35	15.11	2.56	0.26	<1.00E-06	Cytoplasm, extracellular exosome, focal adhesion, plasma membrane, nucleus, lysosomal membrane, late endosome membrane
HNRNPK	Heterogeneous nuclear ribonucleoprotein K	5.09	3.49	28.38	5.39	0.18	<1.00E-06	Cytoplasm, nucleus, podosome, extracellular exosome, membrane, focal adhesion, chromatin
KRT20	Keratin_ type I cytoskeletal 20	0.35	0.39	6.93	1.24	0.05	<1.00E-06	Cytoplasm, cytoskeleton, intermediate filament cytoskeleton
NKX2-6	Homeobox protein Nkx-2.6	0.24	0.18	1.65	0.36	0.14	2.00E-06	Nucleus
RPS16	40S ribosomal protein S16	22.42	6.76	70.57	13.52	0.32	3.00E-06	Cytoplasm, nucleolus, nucleoplasm, extracellular exosome, focal adhesion, membrane
RPRD2	Regulation of nuclear pre-mRNA domain-containing protein 2	1.34	0.54	20.31	4.11	0.07	3.00E-06	nucleoplasm, transcription perinitiation complex
PPP1R12B	Protein phosphatase 1 regulatory subunit 12B	1.11	0.41	6.33	1.37	0.17	5.00E-06	Cytoskeleton, stress fiber

Table S3-5. Top 10 upregulated DEPs through snapshot imprinting analysis comparing H522 to BEAS-2B cell line.

Gene Name	Protein Name	H522 Average Amount (fmol)	STD	BEAS-2B Average Amount (fmol)	STD	Fold Change	P value	Location
HAUS5	HAUS augmin-like complex subunit 5	0.59	0.19	0.02	0.05	26.72	3.90E-05	Centrosome
RGS3	Isoform 2 of Regulator of G-protein signaling 3	0.10	0.03	0.004	9.00E-19	23.08	5.80E-05	Cytoplasm, nucleus, plasma membrane
METTL3	N6-adenosine-methyltransferase 70 kDa subunit	3.33	1.35	0.002	0.004	1345.27	2.15E-04	Nucleus, nucleus speckle, cytoplasm, golgi apparatus
SLX4IP	Protein SLX4IP	1.09	0.44	0.03	0.01	35.89	2.50E-04	Unknown
MYO18B	Unconventional myosin-XVIIIb	2.57	1.08	0.10	0.06	26.87	3.42E-04	Cytoplasm, nucleus, sarcomere
HECW2	E3 ubiquitin-protein ligase HECW2	0.18	0.08	0.02	0.05	9.27	3.46E-04	Cytoplasm, spindle
FRMPD1	FERM and PDZ domain-containing protein 1	0.19	0.08	0.001	7.00E-1	315.87	4.20E-04	Cytoplasm, plasma membrane, cytoskeleton
PPP1R26	Protein phosphatase 1 regulatory subunit 26	0.64	0.21	0.22	0.14	2.95	4.93E-04	nucleolus
CABP1	Calcium-binding protein 1	2.74	1.25	0.06	0.04	45.40	5.02E-04	Cytoplasm, cytoskeleton, perinuclear region, plasma membrane, golgi apparatus, extracellular space
KIF1B	Isoform 3 of Kinesin-like protein KIF1B	1.03	0.48	0.0002	0.0002	6543.56	5.17E-04	Mitochondrion

Table S3-6. Top 10 downregulated DEPs through snapshot imprinting analysis comparing H522 to BEAS-2B cell line.

Gene Name	Protein name	H522 Average Amount (fmol)	STD	BEAS-2B Average Amount (fmol)	STD	Fold Change	P value	Location
KRT17	Keratin_ type I cytoskeletal 17	8.81	4.29	27.05	3.60	0.33	<1.00E-06	Cytoplasm, cytosol, cytoskeleton,
ALMS1	Alstrom syndrome protein 1	9.12	5.15	31.71	5.44	0.29	<1.00E-06	Cytoplasm, centrosome, cilium basal body, spindle pole, mitochondrion, nucleoplasm
ANXA6	Annexin A6	3.49	2.68	15.11	2.56	0.23	<1.00E-06	Cytoplasm, extracellular exosome, focal adhesion, plasma membrane, nucleus, lysosomal membrane, late endosome membrane
HNRNPK	Heterogeneous nuclear ribonucleoprotein K	4.29	2.82	28.38	5.39	0.15	<1.00E-06	Cytoplasm, nucleus, podosome, extracellular exosome, membrane, focal adhesion, chromatin
KRT20	Keratin_ type I cytoskeletal 20	0.68	1.07	6.93	1.24	0.10	<1.00E-06	Cytoplasm, cytoskeleton, intermediate filament cytoskeleton
RPS16	40S ribosomal protein S16	19.29	10.70	70.57	13.52	0.27	1.00E-06	Cytoplasm, nucleolus
KLF3	Krueppel-like factor 3	3.37	1.96	15.97	3.30	0.21	1.00E-06	Nucleus, nucleoplasm
MTAP	S-methyl-5'-thioadenosine phosphorylase	5.13	3.49	17.35	2.03	0.30	3.00E-06	Cytoplasm, Nucleus, extracellular exosome
PPP1R12B	Protein phosphatase 1 regulatory subunit 12B	1.22	0.60	6.33	1.37	0.19	3.00E-06	Cytoskeleton, stress fiber
RPRD2	Regulation of nuclear pre-mRNA domain-containing protein 2	1.44	0.52	20.31	4.11	0.07	3.00E-06	nucleoplasm, transcription perinitiation complex
